# Pathophysiology and Management of Amphetamine-Related Psychiatric Disorders

**DOI:** 10.1192/j.eurpsy.2024.854

**Published:** 2024-08-27

**Authors:** T. Jupe, N. Stamoulis, A. Varsamis

**Affiliations:** Psychiatric Hospital of Attica, Athens, Greece

## Abstract

**Introduction:**

Amphetamines may induce symptoms of psychosis very similar to those of acute schizophrenia spectrum psychosis. This has been an argument for using amphetamine-induced psychosis as a model for primary psychotic disorders. To distinguish the two types of psychosis on the basis of acute symptoms is difficult. However, acute psychosis induced by amphetamines seems to have a faster recovery and appears to resolve more completely compared to schizophrenic psychosis.

**Objectives:**

The objectives of this e-poster is to identify the pathophysiology of amphetamine-related psychiatric disorders and outline the available treatment and management options for amphetamine-related psychiatric disorders.

**Methods:**

A bibliopgraphical review was performed using PubMed platform. All relevant articles were found using the keywords: psychotic episode, amphetamines, pathophysiology and menagement.

**Results:**

Amphetamines inhibit monoamine (dopamine, norepinephrine, epinephrine, serotonin) reuptake, leading to increased monoamine concentrations in the neuronal synapse. Amphetamines can also lead to increased monoamines in the cytosol by interactions with vesicular monoamine transporter 2. Dopamine and norepinephrine release in the nucleus accumbens results in a feeling of euphoria and a reward feedback loop, which may result in addiction. Studies also suggest increased dopaminergic pathways lead to glutamate excesses in the cerebral cortex, altering the function of cortical GABAergic neurons. This damage leads to dysregulation of glutamate in the cerebral cortex, a precursor to psychosis. Prior psychiatric studies have found that GABAergic cortical dysfunction seems to relate to schizophrenia. Generally, acutely agitated psychotic patients are treated with intravenous benzodiazepines (lorazepam, diazepam, or midazolam) as first-line agents. However, if a second-line agent is needed, antipsychotic medicines like risperidone, haloperidol, ziprasidone, and olanzapine have been successful in managing amphetamine-associated psychosis. Lipophilic beta-blockers, such as metoprolol and labetalol, have also been used successfully to resolve agitation and hyperadrenergic vital signs.

**Conclusions:**

Compared to schizophrenic psychosis, amphetamine-induced acute psychosis induced appears to demonstrate a more rapid recovery. It also seems to resolve with substance abstinence; however, this recovery may be incomplete.

**Disclosure of Interest:**

None Declared

